# N6-methyladenosine modifications in maternal-fetal crosstalk and gestational diseases

**DOI:** 10.3389/fcell.2023.1164706

**Published:** 2023-03-16

**Authors:** Suqi Wu, Ketong Liu, Bingyan Zhou, Suwen Wu

**Affiliations:** ^1^ First Clinical Medical College, Zhejiang Chinese Medical University, Hangzhou, China; ^2^ Department of Obstetrics, Obstetrics and Gynecology Hospital of Fudan University, Shanghai, China; ^3^ Hubei Clinical Center of Hirschsprung’s Disease and Allied Disorders, Department of Pediatric Surgery, Tongji Hospital, Tongji Medical College, Huazhong University of Science and Technology, Wuhan, China

**Keywords:** N6-methyladenosine, maternal-fetal crosstalk, gestational diseases, epigenetics, fetal growth

## Abstract

As a medium among pregnant women, environment and fetus, placenta owns powerful and delicate epigenetic processes to regulate gene expression and maintain cellular homeostasis. N6-methyladenosine (m^6^A) is the most prevalent modification that determines the fate of RNA, and its dynamic reversibility indicates that m^6^A may serve as a sensitive responder to environmental stimuli. Emerging evidence suggests that m^6^A modifications play an essential role in placental development and maternal-fetal crosstalk, and are closely related to gestational diseases. Herein, we summarized the latest techniques for m^6^A sequencing and highlighted current advances of m^6^A modifications in maternal-fetal crosstalk and the underlying mechanisms in gestational diseases. Therefore, proper m^6^A modifications are important in placental development, but its disturbance mainly caused by various environmental factors can lead to abnormal placentation and function with possible consequences of gestational diseases, fetal growth and disease susceptibility in adulthood.

## 1 Introduction

The placenta acts as a medium for pregnant women and fetuses, enabling the exchange of nutrients, gas and also waste productions, protecting fetuses from maternal immune attack, and also secreting hormones and factors to support fetal growth. The defects of placentation will result in a range of gestational diseases (Jauniaux et al., 2006; Ananth, 2014; Roberts, 2014; Ashraf et al., 2021), such as miscarriage, preterm birth, gestational diabetes mellitus, pre-eclampsia and fetal growth restriction. Placental development during the whole gestation involves a variety of cellular function changes and transformations (Caniggia et al., 2000; Pringle et al., 2010). This rapid development makes the prenatal period particularly vulnerable because of the complicated trajectory of cells and environmental perturbations. To maintain placental homeostasis, several epigenetic mechanisms have been highlighted to drive short- and long-term gene expression changes (Nelissen et al., 2011), including DNA methylation (Koukoura et al., 2012; Lorincz and Schubeler, 2017), histone modifications (Meister et al., 2021), non-coding RNAs (Du et al., 2021; Zhang et al., 2021; Zarkovic et al., 2022), and also RNA methylation (Mu et al., 2022). As one of the most prevalent internal modifications in RNAs, N6-methyladenosine (m^6^A) extensively regulate RNA metabolism (Jia et al., 2022). Because of its involvement in RNA-related bioprocesses, m6A is essential in decision of RNA fate and plays an irreplaceable role in cell proliferation, differentiation, stress responses and other activities (Wang et al., 2014a; Zhang et al., 2017a; Zhang et al., 2017b). A recent study based on multi-organ m^6^A sequencing showed that placental tissues are rich in m6A modifications, and tissue specific m^6^A may be related to unique biofunctions (Xiao et al., 2019; Zhang et al., 2020a). Some other studies also reported the relationship between m^6^A dysregulation and gestational diseases (Li et al., 2019; Taniguchi et al., 2020; Qiu et al., 2021; Wang et al., 2021; Xu et al., 2021; Zhang et al., 2021). Therefore, in this review, we summarize the role of m^6^A in placental development and gestational diseases, and provide novel insights for underling mechanisms during these processes.

## 2 Core regulators of m^6^A

**FIGURE 1:**
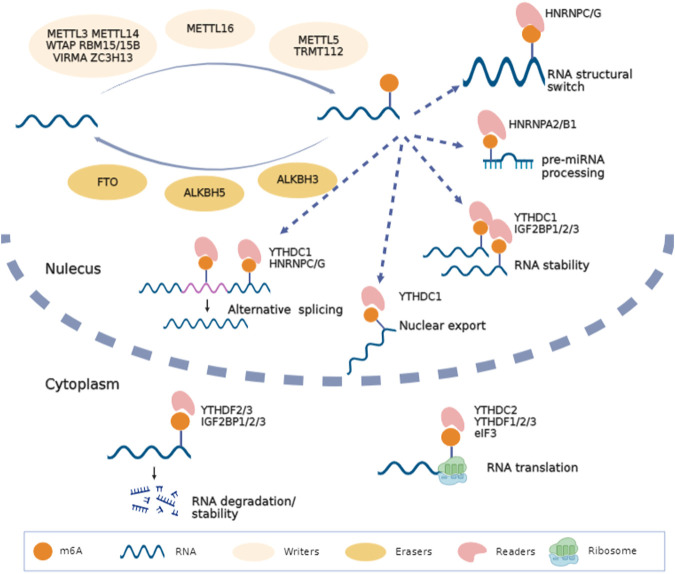
Biofunctions of m^6^A in RNA metabolism. RNA m^6^A is methylated under the action of methyltransferases (METTL3, WTAP, METTL14, RBM15/15B, VIRMA, ZC3H13, METTL16, METTL5 and TRMT112), demethylated mediated by demethylases (FTO, ALKBH5 and ALKBH3) and recognized by binding proteins (YTHDC1/2, YTHDF1/2/3, IGF2BP1/2/3, HNRNP and eIF3). This figure was drawn using BioRender (https://www.biorender.com).

Over 100 kinds of chemical modifications of RNA have been identified in organisms, including protein-coding RNAs and non-coding RNAs (Saletore et al., 2012). Among them, m6A modification is the most abundant internal methylation of mRNA in eukaryotes, which typically accounts for 0.1%–0.4% of total RNA adenosines (Dominissini et al., 2012). In mammalian cells, m6A is mostly enriched in the 3′-untranslated regions, near stop codons and long exons, and with a consensus sequence of RRACH (R = G or A; H = A, C or U) (Meyer et al., 2012). Transcriptome-wide profile of m^6^A through m^6^A antibody-based immunoprecipitation followed by high-throughput sequencing has validated that m^6^A modification may regulate more than 7000 mRNAs in human transcripts and also lncRNAs, miRNAs and circRNAs (Dominissini et al., 2012; Meyer et al., 2012; Zhao et al., 2014). Similar to DNA and histone modifications, m^6^A is dynamic and reversible. RNA m^6^A modifications can be methylated under the action of methyltransferases and demethylated mediated by demethylases (Table 1). Then, binding proteins can recognize this specific modification and regulate RNA metabolism (Bokar et al., 1994; Liu et al., 2014; Ping et al., 2014). High conserved m^6^A is widely involved in the decision of RNA fate, including alternative splicing, transportation, stability, and translation (Figure 1).

**TABLE 1:** The biofunctions of m^6^A key enzymes in RNA metabolism.

	Factors	Biofunctions	References
Writer	METTL3	Component of methyltransferase complex (MTC); m^6^A catalyst	Liu et al. (2014), Wang et al. (2016)
	METTL14	Component of MTC; stabilize MTC and identify m^6^A motif	Liu et al. (2014), Wang et al. (2016)
	WTAP	Component of MTC; recruit METTL3 and METTL14 into nuclear speckles	Ping et al. (2014)
	RBM15/15B	Component of MTC; direct MTC to specific RNA sites	Knuckles et al. (2018), Zhao et al. (2022)
	KIAA1429/VIRMA	Component of MTC; mediate preferential m^6^A in 3′UTR and near stop codon	Yue et al. (2018), Zhang et al. (2022)
	ZC3H13	Component of MTC; keep MTC in the nuclear speckles	Knuckles et al. (2018), Wen et al. (2018)
	METTL16	m^6^A catalyst	Shima et al. (2017), Warda et al. (2017)
	METTL5	m^6^A catalyst for 18S rRNA	Huang et al. (2022), Sepich-Poore et al. (2022)
	TRMT112	Component of METTL5-TRMT112 MTC; stabilize METTL5	Sepich-Poore et al. (2022)
	ZCCHC4	m^6^A catalyst for 28S rRNA	Ren et al. (2019), Pinto et al. (2020)
Eraser	FTO	Remove m^6^A	Hess et al. (2013), Zhao et al. (2014), Qiu et al. (2021)
	ALKBH5	Remove m^6^A	Tang et al. (2018)
	ALKBH3	Remove m^6^A	Ueda et al. (2017)
Reader	YTHDC1	Alternative splicing; nuclear export; RNA stability	Xiao et al. (2016)
	YTHDC2	Promote translation initiation	Yuan et al. (2022)
	YTHDF1	Promote translation initiation	Wang et al. (2022c)
	YTHDF2	Promote translation elongation and RNA decay	Wang et al. (2015)
	YTHDF3	Promote translation initiation and RNA decay	Chang et al. (2020)
	IGF2BP1/2/3	Promote the RNA stability and translation	Sun et al. (2022)
	HNRNPA2/B1	Promote pre-miRNA processing	Alarcon et al. (2015), Klinge et al. (2019)
	HNRNPC/G	Switch structure and splicing	Huang et al. (2021)
	eIF3	Enhance protein synthesis	Choe et al. (2018)

### 2.1 Writers of m^6^A

Installation of m^6^A can be catalyzed by a methyltransferase complex (MTC) composed of several proteins, or through MTC-independent manners. Methyltransferase-like 3 (METTL3) is the most important component of MTC, with an internal S-adenosyl methionine (SAM)-binding domain and catalyzes methyl group transfer through its highly active methyltransferase domain (Liu et al., 2014; Wang et al., 2016). METTL14, another active component of MTC, is co-localized in nuclear speckles with METTL3 and stabilizes the structure of MTC and identifies specific RNA sequence (Liu et al., 2014; Wang et al., 2016). Other auxiliary proteins are also essential for the stability of MTC, and Wilms tumor 1-associated protein (WTAP) is the first identified protein which recruits METTL3-METTL14 heterodimer into nuclear speckles (Ping et al., 2014). Moreover, RNA-binding motif protein 15 (RAB15/15B) and Vir like m^6^A methyltransferase associated (VIRMA/KIAA1429) can direct MTC to specific RNA sites for m^6^A modification (Knuckles et al., 2018; Yue et al., 2018). Additionally, zinc finger CCCH-type containing 13 (ZC3H13) interacts with WTAP to keep MTC in nuclear speckles (Knuckles et al., 2018; Wen et al., 2018). Among these components, except METTL3, all other factors lack methyltransferase activity. In addition, another MTC of METTL5 and tRNA methyltransferase activator subunit 112 (TRMT112) has been identified as a m^6^A methyltransferase for 18S rRNA (Huang et al., 2022; Sepich-Poore et al., 2022).

METTL16 and Zinc finger CCHC-type Containing 4 (ZCCHC4) are newly identified independent RNA methyltransferases. METTL16 contains two structural domains and catalyzes m^6^A in the 3′UTR in mRNA and on A43 of U6 snRNA (Shima et al., 2017). METTL16 also plays an important role in RNA splicing, which targets for pre-mRNAs and non-coding RNAs (Warda et al., 2017). In addition, ZCCHC4 contains an N-terminal specific zinc finger domain and a C-terminal CCHC domain for RNA binding and catalyzes m^6^A in 28S rRNA (Ren et al., 2019; Pinto et al., 2020).

### 2.2 Erasers of m^6^A

Fat mass and obesity-associated protein (FTO) is the first reported m^6^A demethylase which was discovered in 2011 (Jia et al., 2011). After that, the second eraser, AlkB homolog 5 (ALKBH5), was discovered (Zheng et al., 2013). Both of them belong to the alpha-ketoglutarate-dependent dioxygenase family and remove the m^6^A modification labeled in mRNAs and ncRNAs through an Fe (II) and α-ketoglutaric acid-dependent manner. FTO is partially located in nuclei and can be recruited to the spliceosome center by its nuclear speckle partner, and then participates in RNA processing. Similarly, ALKBH5 is verified to co-localize in nuclear speckles with other RNA processing factors and is essential in RNA synthesis, transport and stability. In addition, ALKBH3 is newly reported to demethylate m^6^A in mammalian tRNA (Ueda et al., 2017; Chen et al., 2019).

### 2.3 Readers of m^6^A

Different downstream biofunctions of RNA m^6^A depend on various RNA binding proteins called m^6^A readers. These readers discovered to date include YT521-B homology (YTH) domain-containing proteins (YTHDF1/2/3 and YTHDC1/2), heterogeneous nuclear ribonucleoproteins (HNRNPA/B/C/G), insulin-like growth factor 2 mRNA binding proteins (IGF2BP1/2/3) and eukaryotic initiation factor 3 (eIF3). Considering the different cellular localizations during RNA metabolism, readers can be categorized as nuclear readers and cytoplasmic readers. The former is more likely to participate in alternative splicing, RNA structural switch and nuclear export (Xiao et al., 2016; Roundtree et al., 2017). Typically, YTHDC1 can recruit serine- and arginine-rich splicing factor 3 (SRSF3) and SRSF10 to promote exon inclusion and skipping (Xiao et al., 2016). YTHDC2 also promotes the nuclear export of m^6^A-modified mRNA through interacting with nuclear RNA export factor 1 (NXF1) and the three prime repair exonuclease (TREX) mRNA export complex (Lesbirel et al., 2018). HNRNPC/G act as nuclear RNA binding proteins and are responsible for pre-mRNA processing; m^6^A modified mRNA alters their local structure and regulate the HNRNPC/G activities to finally affect the abundance as well as alternative splicing (Liu et al., 2015; Liu et al., 2017). In addition, IGF2BPs can identify m^6^A and further promote RNA stability and translation (Sun et al., 2022). Some evidence shows that m^6^A in 5′UTR can recruit eIF3 directly and then increase the efficacy of cap-independent translation (Meyer et al., 2015). Hence, the final fate of RNA depends on its m^6^A sites and the type of readers recognizing the effective m^6^A motif (Figure 1).

## 3 Detection techniques for RNA m^6^A modifications

The development of high-throughput m^6^A sequencing significantly promote the exploration in epitranscriptomics field, especially in the distribution, proportion and function of m6A in transcriptome wide (Table 2).

**TABLE 2:** Detection methods of m^6^A.

Techniques	Date	Dependence	Resolution	Quantification	Others	References
MERIP-seq/m^6^A-seq	2012	m^6^A antibody	100–200 nt	No.	Most used method. Large amount of initial RNA. Full commercialization	Dominissini et al. (2012), Meyer et al. (2012)
m^6^A-LAIC-seq	2016	m^6^A antibody; ERCC RNA Spike-In Mix	Gene level	Yes	Cannot obtain m^6^A positions and fractions	Molinie et al. (2016)
PA-m^6^A-seq	2015	m^6^A antibody; UV365; 4SU	23–30 nt	No.	Work in cultured cell lines	Chen et al. (2015)
m^6^A-CLIP/miCLIP	2015	m^6^A antibody; UV254	Single base	No.	An indirect way to analyze m^6^A positions. Low crosslinking yield	Ke et al. (2015), Linder et al. (2015)
m^6^ACE	2019	UV254; exonuclease XRN1	Single base	Yes	Directly map transcriptome-wide m^6^A	Koh et al. (2019)
m^6^A-REF-seq/MAZTER-seq	2019	MazF	Single base	Yes	Cover a limited proportion of m^6^A sites (16%–25%)	Garcia-Campos et al. (2019), Zhang et al. (2019)
DART-seq	2019	APOBEC1- YTH protein	Single base	No.	Low requirement of initial RNA. Affected by vector transfection. Cover a limited proportion of m^6^A sites	Meyer (2019)
scDART-seq	2022	APOBEC1- YTH protein	Single base	No.	Single cell detection	Tegowski et al. (2022)
m^6^A-SEAL	2020	FTO	100–200 nt	No.	Full commercialization. Expand the application for m^6^A imaging	Wang et al. (2020)
m^6^A-label-seq	2020	a^6^A antibody	Single base	No.	Work in cultured cell lines. Low incorporation efficacy of a^6^A	Shu et al. (2020)
m^6^A-SAC-seq	2022	MjDim1	Single base	Yes	Low requirement of initial RNA.	Hu et al. (2022)

### 3.1 MERIP-seq and m^6^A-seq

In 2012, MERIP-seq and m^6^A-seq were the first high-throughput sequencing methods which were in dependently developed by two laboratories (Dominissini et al., 2012; Meyer et al., 2012). The theory and experimental methods of these two techniques are similar. The RNA fragments containing m^6^A modified sites are incubated and enriched by m^6^A antibody, and then they are subjected to the library preparation and sequencing. Because of simple operation and reagents, this technique is successfully applied to reveal the transcriptome methylation across different species (Schwartz et al., 2013; Luo et al., 2014; Ma et al., 2017). However, high-resolution site information is difficult to obtain because of the limited size of fragmented RNA which could only be about 100–200 nt. Antibody-based IP systems also requires a large amount of initial RNA (400 µg mRNA or 2.5 mg total RNA) and cannot identify precise m^6^A sites (Dominissini et al., 2012). In next year, Dominissini et al. improved the methods and reduced the initial requirement of total RNA (5 µg mRNA or 300 µg total RNA) (Dominissini et al., 2013), which expanded the application of this technology. MeRIP-seq is widely used to uncover the dynamic modifications in physiological and pathological processes (Lence et al., 2016; Zhang et al., 2017a; Zhao et al., 2017).

In 2016, m^6^A-LAIC-seq (m^6^A-level and isoform-characterization sequencing) was developed to quantify m^6^A stoichiometry on the transcriptome level (Molinie et al., 2016). In general, the experimental steps of m^6^A-LAIC-seq are similar to MERIP-seq and m^6^A-seq. There are two main differences: excess m^6^A antibodies are used to enrich full-length transcripts rather than RNA fragments; equal amount of ERCC RNA Spike-In Mix is used for normalization and calculation of m^6^A levels. This technique also provides new insights into the dynamic range and isoform complexity of the m^6^A epitranscriptome. However, use this method alone cannot obtain m^6^A positions and fractions, and the combination with other methods may compensate this drawback.

### 3.2 m^6^A-seq based on photo-crosslinking

In 2015, Chuan He et al. developed a photo-crosslinking-assisted m^6^A sequencing strategy (PA-m^6^A-seq) (Chen et al., 2015). In this technique, the mixture of m^6^A antibody and purified RNA incorporated with 4-thiouridine (4SU) is exposed to 365 nm UV light to trigger the covalent crosslinking between antibody and m^6^A modified RNA. Then, crosslinked RNA is digested to around 30 nt, which greatly improve the resolution of sequencing data. Incorporation of 4SU can induce a T-to-C transition which serves as hallmarks of nearby m^6^A and further improves the resolution to 23 nt. However, this method only works in cultured cell lines and may get false negative results when 4SU is not involved in m^6^A sites.

m^6^A-CLIP (Ke et al., 2015) and miCLIP (m^6^A individual-nucleotide-resolution cross-linking and immunoprecipitation) (Linder et al., 2015) are similar to PA-m^6^A-seq. The RNA-antibody mixture is crosslinked by 254 nm UV light, and then RNA is released by proteinase K. Mutations (C-to-T transition) and truncations induced by crosslink are introduced during reverse transcription, and their patterns are predictable which helps elevating single-nucleotide resolution of m^6^A. However, these techniques still represent an indirect way to analyze m^6^A positions and suffer from a low crosslinking yield. They are also difficult to precisely identify m^6^A sites and detect m^6^A clusters (Shu et al., 2020).

Based on photo-crosslinking, m^6^ACE (m^6^A-crosslinking-exonuclease-sequencing) combines exonuclease to improve the sequencing resolution (Koh et al., 2019). In this technique, UV-crosslinked RNA with m^6^A antibody is exempt from digestion by exonuclease XRN1. After sequencing, XRN1 treated reads start exactly at the m^6^A location when compared to input reads. Hence, m^6^ACE-seq can directly map transcriptome-wide m^6^A at quantitative single-base-resolution. This method is also useful to explore the target m^6^A modifications which are uniquely mediated by specific m^6^A regulators. However, the use of splice fragments with specific molecular identification to decrease the deviation may cause a higher cost.

### 3.3 m^6^A-seq based on endoribonuclease

*Escherichia coli* MazF toxin is an endoribonuclease sensitive to m^6^A which specifically cleaves unmodified ACA-sequence rather than m^6^ACA sites (Imanishi et al., 2017). m^6^A-REF-seq (Zhang et al., 2019) and MAZTER-seq (Garcia-Campos et al., 2019) use MazF enzyme to directly fragment RNA, cleaving the motif sequence ACA from the 5’ side of first A and leaving the m^6^ACA motif intact. This method can identify single base m^6^A at the transcriptome level, and also quantify the methylation level of each m^6^A site by calculating the ratio of reads with internal ACA reverse reads split at the motif. However, this technique can only cover a limited proportion of m^6^A sites because the ACA motif MazF recognized accounts for 16% of total m^6^A sites in mammals and 25% in yeast (Garcia-Campos et al., 2019).

### 3.4 m^6^A-seq based on m^6^A binding proteins

In 2019, Kate D Meyer presented DART-seq (deamination adjacent to RNA modification targets) using APOBEC1-YTH recombinant protein (Meyer, 2019). APOBEC1 is a cytidine deaminase which can induce C-to-U transition. Because YTH domain can specifically recognize m^6^A site, the deaminase activity of APOBEC1-YTH is mainly induced by YTH. Meanwhile, a mutant APOBEC1-YTH without m^6^A binding function is transfected into cells as a negative control. DART-seq can identify m^6^A using as little as 10 ng of initial RNA, and facilitate exploration of m^6^A in limited samples. Even more surprising, DART-seq can be coupled with single cell isolation and achieves single-cell m^6^A detection (Tegowski et al., 2022). scDART-seq distinguishes cellular subpopulations based on m^6^A signatures rather than gene expression, revealing abundant m^6^A features in cellular functions. However, the efficacy of vector transfection during cell culture limits the application of DART-seq on tissues samples. YTH domain only recognize around 60% of total m^6^A sites, which may miss several modification sites.

In 2020, m^6^A-SEAL was developed which relies on FTO activity (Wang et al., 2020). In principle, FTO recognizes and binds to m^6^A sites, and then converts m^6^A into N6-hydroxymethyladenosine [hm (Pringle et al., 2010) A]. After treatment with DTT, hm^6^A is converted into a stable chemical N6-dithiolsitolmethyladenosine [dm (Pringle et al., 2010) A] which can be tagged with biotin for streptavidin enrichment and sequencing. Moreover, optimization of the FTO oxidation, m^6^A-SEAL can also be used to specifically detect cap m^6^A. When compared to other m^6^A sequencing methods and specific validation methods, m^6^A-SEAL shows great sensitivity, specificity and reliability for transcriptome-wide m^6^A detection. Considering the rich tagging ability, it may expand the application of m^6^A, especially in m^6^A enrichment and imaging.

### 3.5 m^6^A-seq based on chemical labeling

m^6^A-label-seq is a metabolic labeling method to map transcriptome-wide m^6^A modifications at single-base resolution through converting stable m^6^A structure into a reactive one (Shu et al., 2020). In this technique, cells are incubated with a methionine analog, Se-allyl-L-selenohomocysteine, to incorporate allyl-group into m^6^A sites and introduce allyladenosine (a^6^A). Then, anti-a (Pringle et al., 2010) A antibody is used to enrich the a^6^A modified mRNA to achieve high coverage of m^6^A target. Under mild conditions, iodine can undergo addition reaction with a^6^A and obtain CycA through spontaneous cyclization. During the reverse transcription, misincorporation at the opposite site in cDNA will occur and m^6^A sites can be detected after sequencing. However, this method only works in cultured cell lines and may also get false negative results because of the low incorporation efficacy of a^6^A. Similar to m^6^A-SEAL (Wang et al., 2020), m^6^A-label-seq offers a new option for m^6^A specific mapping, but lacks stoichiometric information.

In 2022, Chuan He et al. developed a new technique based on selective allyl chemical labeling, m^6^A-SAC-seq (Hu et al., 2022), which can map the transcriptome-wide m^6^A at single-nucleotide resolution and also achieve stoichiometric information. MjDim1, a member in Dim1/KsgA family, is used to transfer the methyl group from allylic-S-adenosyl-L-methionine (SAM) to adenosines, converting m^6^A into allyl-modified m^6^A (N6-allyl, N6-methyladenosine or a^6^m^6^A). a^6^m^6^A can undergo cyclization following I2 treatment, and then mutations will occur in the process of reverse transcription. The m^6^A positions can be detected according to the mutation sites, and the accurate proportion can be calculated by converting the mutation rate through the standard curve. This method requires only 30 ng of poly(A) or rRNA-depleted RNA which largely expand the application in limited samples.

## 4 RNA m^6^A act as sensitive effectors and regulators in maternal-fetal crosstalk

**FIGURE 2:**
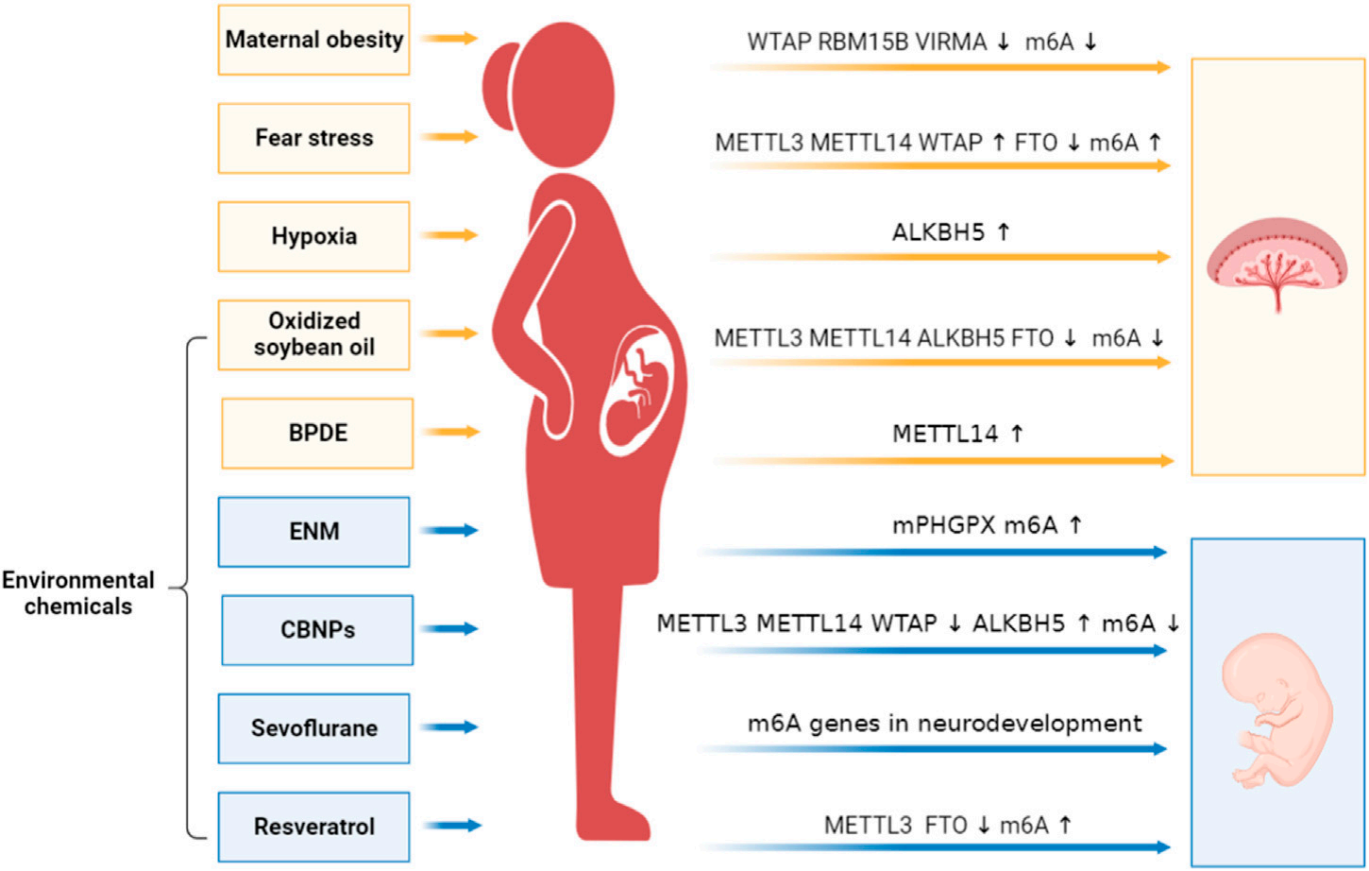
RNA m^6^A act as sensitive effectors and regulators in maternal-fetal crosstalk. Several environmental factors act on the mothers, and affect the m^6^A modifications in placentas (orange) and fetal tissues (blue). Alterations of m^6^A modifications regulate the expression of hub genes and disturb the placental and fetal development. This figure was drawn using BioRender (https://www.biorender.com).

Placentation is a physiological process with dynamic and precise regulation in time and space. Epigenetic processes which drive short- and long-term gene expression changes are particularly powerful and delicate in placentas. RNA m^6^A methylomes across fetus and adult tissues depicted the dynamic m^6^A methylation across different tissue types, covering both broadly or tissue-specifically m^6^A sites (Xiao et al., 2019; Zhang et al., 2020a). In agreement with previous studies, the m^6^A modifications in placental tissues are mostly enriched around stop codons, with a consensus motif of RRACH, indicating the high conservation of m^6^A in human placentas (Taniguchi et al., 2020; She et al., 2022). However, abundant tissue-differential m^6^A peaks are identified in placenta which is higher than that in brain, heart, kidney and other tissues (Xiao et al., 2019). More than half of placental specific m^6^As is located in introns, suggesting the potentially higher activity of RNA splicing in placentas (Xiao et al., 2019). Hence, the detailed mechanism of RNA m^6^A in placentation and maternal-fetal crosstalk needs to be further understood (Figure 2).

### 4.1 RNA m^6^A in maternal obesity and stress

The growing prevalence of maternal obesity or overweight shows a higher risk of abnormal fetal growth and failed placentation. In obese pregnant women, global m^6^A levels are decreased in placental tissues, along with decreased expression of WTAP, RBM15B and VIRMA; while, the obesity also triggers genome-wide DNA hypermethylation, especially in 5-methylcytosine (5 mC) (Shen et al., 2022). In pigs, maternal obesity is associated with low birth weight (LBW) of piglets. In LBW placentas derived from sows with obese pregnancy, the protein level of FTO is decreased, with an elevated m^6^A level (Song et al., 2018). FTO demethylates m^6^A of PPARγ, VEGFA, ABHD5, and GPR120, and the expression levels of these genes in both mRNA and protein are decreased in LBW placentas (Song et al., 2018). Therefore, maternal obesity may regulate multifaceted gene network to affect normal placentation and fetal growth (Song et al., 2018; Shen et al., 2022).

External stimuli such as fear stress can cause significant harm to pregnant women and increase the risk of fetal malformations and placental structural alterations (Tsui et al., 2006; Mizrak and Kabakci, 2021). In pregnant rats, fear stress during gestation significantly reduces placental weight and offspring viability (Wang et al., 2022a). Stress also increases the expression levels of METTL3, METTL14 and WTAP, decreases the level of FTO, and leads to a higher overall m^6^A level in placental tissues (Wang et al., 2022a). While, stress has no obvious change in the distribution of m^6^A-enriched regions and motif. Based on MeRIP-seq data, fear stress mainly affects m^6^A-modified genes involved *in utero* embryonic development, protein stabilization, angiogenesis and embryonic digit morphogenesis (Wang et al., 2022a). Similar changes are also found in brain development and adult neurons (Widagdo et al., 2016; Walters et al., 2017). Fear stress can alter the levels of METTL3 and FTO, thereby affecting m^6^A modifications and synaptic plasticity (Widagdo et al., 2016; Walters et al., 2017).

### 4.2 RNA m^6^A in placental hypoxia

In the first trimester, the presence of extravillous trophoblast plugs in the spiral arteries changes the level of oxygen in placental circulation, inducing trophoblast differentiation and invasion, and arterial transformation (Caniggia et al., 2000; Iriyama et al., 2015; Albers et al., 2019). Disturbance of this orderly process may lead to abortion and ischemic placental diseases (Ananth, 2014; Roberts, 2014). Hypoxia treatment can upregulate the expression level of ALKBH5 in trophoblast cells, and it also promotes ALKBH5 to translocate from nuclear to cytoplasm to demethylate m^6^A-modified SMAD1/5 mRNA. High level of SMAD1/5 further activates TGF-β signaling pathway and increases the expression of MMP9 and ITGA1 to promote the cellular viability of trophoblast (Zheng et al., 2022). Inhibition of ALKBH5 promotes cellular viability and inhibits cell apoptosis, oxidative stress in hypoxia/reoxygenation treated trophoblast cells, and finally alleviates preeclampsia-like symptoms in pregnant mice. ALKBH5 knockdown facilitates the m^6^A modification of PPARG mRNA and further activates the Wnt/β-catenin pathway (Guo et al., 2022). Consistent with other reports in cancers (Chen et al., 2020a; Dong et al., 2021; Sun et al., 2023), hypoxia-responsive ALKBH5 may be an important regulator in trophoblast activity and placental development.

### 4.3 RNA m^6^A in maternal exposure to environmental chemicals

Oxidized oil or lipid oxidation products, especially maternal oxidative stress damage, have toxicological effects of placenta and fetus. The placental tissues from rat fed with oxidized soybean oil (OSO) had no significant change in the distribution of m^6^A-modified regions and motif. However, the mRNA expression levels of *Mettl3*, *Mettl14, Alkbh5* and *Fto* are decreased in placentas after OSO ingestion, with a lower global m^6^A level. The placental genes with differential m^6^A levels are related to nutrient metabolic process and hormone activity (Wang et al., 2022b). In addition, exposure to BPDE [benzo(a)pyrene-7,8-dihydrodiol-9,10-epoxide], the metabolite of Benzo(a)pyrene (BaP) which is one representative of PAHs (polycyclic aromatic hydrocarbons), upregulates the expression level of m^6^A-modified lnc-HZ01 and inhibits the proliferation of trophoblast cells. More interestingly, m^6^A modified lnc-HZ01 upregulates the expression level of MXD1, and the latter promotes the transcription of METTL14 which in turn catalyzes m^6^A in lnc-HZ01, forming a positive feedback loop to inhibit trophoblast viability (Xu et al., 2021).

### 4.4 RNA m^6^A inheritance over generations

Environmental chemicals can not only disturb the normal progression of placental development and affect intrauterine growth, but also increase the long-term risk of offspring. Epigenetics is a possible link between the environment and fetal growth, and RNA m^6^A is also inherited across generations. Maternal ingestion of oxidized soybean oil (OSO) during gestation and lactation disturbs the homeostasis of DNA/RNA methylation and negatively affects the placental function and intestinal development in offspring, with a reduced height of villi and a lower level of anti-inflammatory factors (Wang et al., 2022b). In addition, maternal engineered nanomaterial (ENM) exposure increases the m^6^A level in the 3′UTR region of mitochondria phospholipid hydroperoxide glutathione peroxidase (mPHGPx) which is an antioxidant enzyme that protects fetal cells from oxidative stress. The m^6^A-modified mPHGPx diminishes antioxidant capacity, damages the mitochondrial function and causes cardiac deficits, which persists into adulthood following ENM exposure through gestation (Kunovac et al., 2021).

Beforehand, RNA m^6^A modification has been reported to be involved in neurodevelopment and neurotransmitter signaling (Du et al., 2019; Shafik et al., 2020). FTO regulates the activity of the dopaminergic midbrain circuitry (Hess et al., 2013), neuronal growth and plasticity (Yu et al., 2018). Environmental stimuli during pregnancy alter the m^6^A modification pattern in fetus and induce the neurodevelopmental impairments. Pregnant mice exposed to carbon black nanoparticles (CBNPs) cause obvious alterations on maternal behaviors, and neurobehavioral and muscular developmental impairments of offspring (Zhang et al., 2020b). Moreover, maternal CBNPs exposure significantly decreases the expression levels of Mettl3, Mettl14 and Wtap, increases the expression level of Alkbh5, and further decreases the global m^6^A level in the cerebral cortex tissues, indicating the potential relation between m^6^A alteration induced by maternal CBNPs exposure and neurodevelopment of offspring at postnatal time (Zhang et al., 2020b). Anesthetic exposure during gestation, especially repeated sevoflurane exposure, significantly damages the memory and learning ability of the offspring mice through inhibiting the axon growth and branching. After repeated sevoflurane exposure, differential m^6^A-modified genes are related to neurodevelopment, especially synapse, main axon and postsynaptic density membrane (Chen et al., 2020b). In addition, a recent study reported that maternal treatment with resveratrol, an anti-inflammatory and synaptic plasticity inductor produced from grapes, can decrease the expression levels of Mettl3 and Fto, as well as increase the global m^6^A levels in adult offspring (Izquierdo et al., 2021). Maternal consumption of resveratrol can prevent cognitive impairment induced by a high-fat diet and this improvement is associated with increased m^6^A levels (Izquierdo et al., 2021).

## 5 RNA m^6^A in great obstetrical syndromes (GOS)

GOS involves a serious of pregnancy-related disorders with a placental component as one part of etiology, including spontaneous abortion, preterm birth, preeclampsia, stillbirth and abnormal fetal growth (Brosens et al., 2011; Prins et al., 2022). Most etiologies arise from events during maternal-fetal exchange, such as nutrients, oxygen, waste products and toxins. Gestational diabetes mellitus is a particular example of pregnancy disorders involving environmental exposome which disturbs the maternal-fetal interaction (Gabbay-Benziv and Baschat, 2015; Valero et al., 2022). Whether and how m^6^A dysregulation contributes to the pathological mechanisms remains to be elucidated (Table 3).

**TABLE 3:** Role of RNA m^6^A in gestational diseases.

Diseases	MeRIP-seq analysis	Factors	Expression	Target	Regulation	Effect in diseases	References
Abortion	Yes	—	—	—	—	DMEGs were mainly involved in the Hippo and Wnt signaling pathways	She et al. (2022)
Abortion	—	ALKBH5	Down	SMAD1/5	ALKBH5 demethylated m^6^A of SMAD1/5 mRNA and enhanced translation, further promoted MMP9 and ITGA1 production	ALKBH5 promoted the trophoblast activity, including proliferation, migration and invasion	Zheng et al. (2022)
Abortion	—	FTO	Down	HLA-G VEGFR MMP	FTO-bound HLA-G, VEGFR and MMP9 RNA was decreased in patients	The downregulation of FTO in the chorionic villi disrupted immune tolerance and angiogenesis at the maternal-fetal interface	Qiu et al. (2021)
		IGF2BP1	Down				
		IGF2BP2	Down				
		METTL3	Up				
		WTAP	Up				
Abortion	—	METTL14	Up	Lnc-HZ01	METTL14-catalyzed m^6^A on lnc-HZ01 enhanced lnc-HZ01 RNA stability	Upregulated lnc-HZ01 inhibited trophoblast cell proliferation and induced miscarriage	Xu et al. (2021)
Abortion	—	ALKBH5	Up	CYR61	ALKBH5 demethylated m^6^A of CYR61 mRNA and decreased the mRNA half-life	Overexpression of ALKBH5 inhibited trophoblast invasion	Li et al. (2019)
GDM	Yes	—	—	—		DMEGs were mainly involved in the fatty acid-metabolism pathway, the peroxisome proliferator-activated receptor signaling pathway, and thyroid hormone signaling pathway	Du et al. (2022a)
GDM	Yes	—	—	—		DMEGs were strongly associated with monocyte infiltration	Du et al. (2022b)
GDM	Yes	METTL14	Down	BAMBI INSR IRS1	The m^6^A levels of the BAMBI, INSR and IRS1 were significantly decreased in GDM, with decreased level of mRNA and protein	Downregulation of m^6^A both in the 3′-UTR and CDS near stop codons of placental mRNAs is involved in GDM development	Wang et al. (2021)
PE	—	METTL14	Up	FOXO3a	METTL14-catalyzed m^6^A on FOXO3a enhanced FOXO3a RNA stability and promoted translation	METTL14 inhibited trophoblast proliferation and invasion, but induced trophoblast autophagy and apoptosis	Fan et al. (2022)
PE	Yes	—	—	—	—	Hub gene-mediated classification is consistent with m^6^A modification clusters for predicting the clinical characteristics of patients with preeclampsia	Li et al. (2022a)
PE	—	ALKBH5	Up	PPARG	ALKBH5 reduced m^6^A levels of PPARG mRNA, and increased PPARG mRNA stability and promoted PPARG translation	ALKBH5 silencing increased cell proliferation, migration, and inhibited cell apoptosis, oxidative stress	Guo et al. (2022)
PE	Yes	METTL14	Up	circPAPPA2	METTL14 increased the level of circPAPPA2 m^6^A methylation and IGF2BP3 maintained circPAPPA2 stability	circPAPPA2 expression was reduced in PE, and knockdown of circPAPPA2 suppressed trophoblast invasion	Zhang et al. (2021)
PE	—	METTL3 HNRNPC1/2	Up Up	HNRNPC1/2	METTL3 knockdown significantly reduced the level of HNRNPC1/2	These may contribute to trophoblast dysfunction in preeclampsia	Gu et al. (2021)
PE	Yes	—	—	SMPD1	m^6^A modification of SMPD1 at the 5′‐UTR promoted protein translation	m^6^A both at the 5′-UTR and in the vicinity of stop codon may play important roles in fetal growth and disease	Taniguchi et al. (2020)

### 5.1 RNA m^6^A in spontaneous abortion (SA)

The known pathological factors of SA include chromosomal abnormalities, maternal infections, endocrine disorders, nutrition, occupational and environmental factors, immunological factors, and inherited thrombophilia. Epigenetics also participates in the pathogenesis, while the detailed mechanism has not been fully understood. Based on MeRIP-seq data of villous tissues from SA, m^6^A peaks are still mainly located in the codding region and near the stop codon, with a consensus sequence of RRACH (She et al., 2022). Differential m^6^A-modified genes are mainly involved in the Hippo and Wnt signal pathways, phosphatase activity regulation and transcription inhibitor activity (She et al., 2022). At the maternal-fetal interface, FTO, IGF2BP1 and IGF2BP2 are decreased in abortion tissues, with a decreased level of FTO-bound HLA-G, VEGFR and MMP9 mRNA (Qiu et al., 2021). Hence, aberrant FTO level changes the m^6^A modifications of hub genes involved in immune tolerance, angiogenesis and trophoblast invasion, indicating the potential pathogenesis of m^6^A in SA. Research focusing on trophoblast function indicates that aberrant m^6^A regulation inhibits the trophoblast activity and leads to SA. Upregulated ALKBH5 demethylates m^6^A of CYP61 mRNA and further decreases the half-life of CYR61 mRNA, inhibiting the trophoblast invasion (Li et al., 2019). ALKBH5 is also sensitive to hypoxic condition which has been supposed to be an important regulator in trophoblast activity, especially in the first trimester (Guo et al., 2022; Zheng et al., 2022). ALKBH5 proteins translocate to the cytoplasm under hypoxia and then demethylates m^6^A-modified SMAD1/SMAD5 mRNA, consequently enhancing the efficacy of translation (Zheng et al., 2022). Trophoblast-specific knockdown of ALKBH5 in mice significantly inhibits the trophoblast invasion and causes abortion (Zheng et al., 2022). In addition, upregulated METTL14 was also reported to inhibit trophoblast proliferation and induce miscarriage through catalyzing m^6^A on lnc-HZ01 and enhancing its RNA stability (Xu et al., 2021).

### 5.2 RNA m^6^A in gestational diabetes mellitus

The increasing prevalence of type 2 diabetes in general and in younger people in particular, has led to an increasing number of affected pregnancies. Its maternal and fetal complications include abortion, malformations, preterm delivery, preeclampsia, etc. Several researchers performed high throughput sequencing and analyzed the data from GEO to explore the underlying mechanism of m^6^A in GDM. Du et al. generated a lncRNA-mediated competitive endogenous RNA (ceRNA) network, and found that hub genes were mainly involved in fatty acid metabolism pathway, which play a role in the development and adverse outcomes of GDM (Du et al., 2022a). Other GDM-associated hormones were also enriched, such as thyroid hormone and oxytocin (Du et al., 2022a). In addition, m^6^A modified genes related to monocyte infiltration were also clinically important in GDM, including CD81, CFH, FABP5, GBP1 etc. (Du et al., 2022b) Decreased level of METTL4 was found in placentas from GDM patients, and the m^6^A levels of BAMBI, INSR and IRS1 which are GDM-related genes were also significantly decreased, with the same change in mRNA and protein levels (Wang et al., 2021). Hence, m^6^A modification may regulate placental metabolism, hormone secretion and immune infiltration in GDM.

### 5.3 RNA m^6^A in preeclampsia and fetal growth restriction

An imposing number of mechanisms have been proposed to explain the occurrence of preeclampsia, and those currently considered important include abnormal trophoblast invasion, immune intolerance, maternal maladaptation to inflammation, and genetic factors including inherited predisposing genes and epigenetics. Nowadays, abundant studies have identified the role of epigenetics in preeclampsia and FGR (Nelissen et al., 2011; Koukoura et al., 2012; Ashraf et al., 2021; Meister et al., 2021), while the potential function and mechanism of m^6^A need to be further explored. The MeRIP-seq data of placentas showed a correlation between higher m^6^A at 5′UTR and small-for-date placentas, and the decreased m^6^A near stop codon was related to heavy-for-date placentas, revealing the different m^6^A modified sites may be important for fetal and placenta growth (Taniguchi et al., 2020). Meanwhile, the m^6^A labeled at 5′UTR promoted the protein translation of SMPD1 mRNA (Taniguchi et al., 2020). Upregulated METTL3 significantly elevated the expression level of HNRNPC1/2, further inducing vitamin D deficiency, trophoblast dysfunction and preeclampsia (Gu et al., 2021). Moreover, METTL14 was upregulated in preeclamptic placentas: its high expression inhibited trophoblast proliferation and invasion, but induced autophagy and apoptosis (Fan et al., 2022). On the mechanism, METTL14 catalyzed m^6^A on FOXO3a and enhanced RNA stability and translation (Fan et al., 2022). Another MeRIP-seq data showed that METTL14 was upregulated and total m^6^A levels of circRNAs were increased in preeclampsia. METTL14 modified m^6^A on circPAPPA2 and the latter was identified by IGF2BP3 to maintain RNA stability (Zhang et al., 2021). The knockdown of circPAPPA2 suppressed trophoblast invasion and the expression level of circPAPPA2 was reduced in preeclampsia. In addition, m^6^A-related bioinformatic analysis was performed to seek the correlation between m^6^A modifications and clinical characteristics of preeclampsia (Li et al., 2022a). Higher m^6^A level was associated with higher maternal age and even a higher rate of FGR.

## 6 Conclusion and future perspectives

The rapid developments in m^6^A sequencing and its relevant methodology have conclusively highlighted the abundant existence and dynamic regulation network of m^6^A modification in RNA metabolism and fate decision, including alternative splicing, translocation, stability and translation (Wang et al., 2014b; Berulava et al., 2015; Tang et al., 2020; Akhtar et al., 2021). The whole-transcriptome m^6^A methylomes across major human tissues depicted the dynamic m^6^A methylation across different tissue types, covering both broadly and tissue-specifically m^6^A sites (Xiao et al., 2019). Moreover, m^6^A modifications have been discovered in various biological functions, including self-renewal and transition of stem cells, cell differentiation, cellular response to stress, hypoxia adaptation, metabolism and secretion, and other bioprocesses (Batista et al., 2014; Lin and Gregory, 2014; Zhang et al., 2017a; Zhang et al., 2017b; Cui et al., 2017; Xu et al., 2017; Frye et al., 2018; Tang et al., 2018; Wang et al., 2018). Hence, m^6^A modification serves as an essential regulator in physiological and pathophysiological conditions. Placental-specific m^6^A modifications are observed when compared with other human tissues, indicating its potentially unique biofunctions in placental development. In this study, we summarize current advances of m^6^A modifications during gestational period and obstetric diseases, and also highlight that m^6^A plays as sensitive effectors and regulators in maternal-fetal interaction.

Considering the existence and regulatory mechanism of epigenetics in placenta, RNA m^6^A may act as a versatile checkpoint that correlates different layers of gene regulation and forms a more complicated regulatory network for cellular homeostasis (Kan et al., 2022). Similar to mRNA, the metabolic processes of non-coding RNAs are also regulated by m^6^A modifications, including RNA synthesis, cellular localization, translation and degradation (Jia et al., 2022). What’s more, RNA m^6^A modifications interact with DNA methylation and histone modifications. ALKBH5 is verified to demethylate m^6^A in DNMT3B mRNA and inhibit the degradation, inducing the pathogenesis of intervertebral disc disorders (Li et al., 2022b). Maternal and environmental factors may affect multiple checkpoints of epigenetics and further induce the pathogenesis of placenta-related diseases.

Nevertheless, our knowledge of m^6^A in placenta, especially placental development and gestational diseases is far from complete. We still have limited information about the physiological changes of m^6^A in developmental placentas which is the basis of placental research. More importantly, m^6^A target RNAs in maternal serum can be a new direction and strategy for the development of novel biomarkers for prenatal diagnosis. Follow-up studies need to address these key issues more specifically.
